# Individualized and Controlled Exercise Training Improves Fatigue and Exercise Capacity in Patients with Long-COVID

**DOI:** 10.3390/biomedicines12112445

**Published:** 2024-10-24

**Authors:** Simon Kieffer, Anna-Lena Krüger, Björn Haiduk, Marijke Grau

**Affiliations:** 1 Institute of Cardiovascular Research and Sports Medicine, Molecular and Cellular Sports Medicine, German Sport University Cologne, 50933 Cologne, Germany; 2 S.P.O.R.T. Institut, Institute of Applied Sports Sciences, 51491 Overath, Germany

**Keywords:** Long-COVID, Post-COVID, fatigue, exercise capacity, 1MSTST, individualized training

## Abstract

(1) Background: Long-term health effects after SARS-CoV-2 infections can manifest in a plethora of symptoms, significantly impacting the quality of life of affected individuals. (2) Aim: The present paper aimed to assess the effects of an individualized and controlled exercise intervention on fatigue and exercise capacity among Long-COVID (LC) patients in an ambulatory setting. (3) Methods: Forty-one (n = 41) LC patients performed an exercise protocol with an individualized control of the patients’ training intensity during the study period based on the individual’s ability to achieve the target criteria. The program was carried out two to three times a week, each session lasted 30 min, and the study parameters were recorded at the beginning of the program, as well as after 6 and 12 weeks, respectively. These included both patient-reported (PCFS questionnaire, FACIT–Fatigue questionnaire) and objective (one-minute sit-to-stand test (1MSTST), workload) outcomes. (4) Results: The exercise training intervention resulted in significant improvements in the FACIT–Fatigue (F(2, 80) = 18.08, *p* < 0.001), 1MSTST ($\chi^{2}$(2) = 19.35, *p* < 0.001) and workload scores ($\chi^{2}$(2) = 62.27, *p* < 0.001), while the PCFS scores remained unchanged. Changes in the workload scores were dependent on the frequency of the completed exercise sessions and were higher in the LC patients with a moderate Post COVID Syndrome Score (PCS) compared to a severe PCS. (5) Conclusions: The individualized and controlled training approach demonstrated efficacy in reducing fatigue and enhancing exercise capacity among outpatient LC patients. However, for complete regeneration, a longer, possibly indefinite, treatment is required, which in practice would be feasible within the framework of legislation.

## 1. Introduction

Sequelae of SARS-CoV-2 infections occur worldwide, with high prevalence and incidence rates in the population, and cause severe health restrictions. Based on current data, a point prevalence of 6–7% can be assumed. Furthermore, the cumulative global incidence of Long-COVID is estimated to affect approximately 400 million individuals, with an anticipated annual economic impact of around $1 trillion, which corresponds to roughly 1% of the global economy [1]. Depending on the time that has passed since the infection, this phenomenon is referred to as Long-COVID or Post-COVID [2]. As an umbrella term, Long-COVID incorporates all long-term health issues that are prevalent more than four weeks after an acute infection, including post-COVID, which describes all symptoms that persist, return, or arise more than twelve weeks after the acute infection [3]. In the following discussion, the term Long-COVID is used to describe the study sample, which includes Post-COVID. The symptoms most frequently reported by those affected by Long-COVID are fatigue, neurological restrictions, and breathlessness [3]. In particular, the main symptom of fatigue, which is defined by a pronounced lack of energy that is not improved by sleep, severely restricts the quality of life for many sufferers and may also lead to the development of accompanying mental illnesses such as depression and anxiety disorders [4,5]. In addition, many sufferers report an intolerance to physical and mental activity, which is associated with an exacerbation of symptoms [6].

Studies that examined the physical and exercise capacity of those affected using functional and performance tests reported below-average test results for most test subjects [7,8]. Furthermore, some authors were able to identify limitations in the heart’s response to exercise in some affected individuals, which is also referred to as chronotropic incompetence [7]. The reported limitations not only cause a high burden of disease for affected individuals, reducing their everyday functions and their ability to work [9]. The healthcare system also faces major (financial) challenges in the face of this “new” disease, for which there is (as yet) no monocausal treatment [10,11]. Therefore, there is a need for cost- and time-effective treatment options, which are easy to access for affected individuals.

As no single cause has yet been found for the multitude of symptoms, symptom-oriented treatment involving various specialist disciplines is at the heart of previous recommendations from national and international experts [10,12,13]. The German recommendation guideline for Long-/Post-COVID pointed out the potential benefits of systematic and individualized exercise training for symptom treatment [10]. The effectiveness of exercise training in the treatment of a variety of chronic diseases has already been empirically proven [14,15,16]. For example, a combination of aerobic and strength training has proven effective in alleviating the symptoms of fatigue in multiple sclerosis and cancer [14,16]. Exercise training has also been shown to increase exercise capacity and quality of life (QoL) across several diseases, including cancer, cardiovascular and musculoskeletal diseases, respiratory conditions, and others [15].

Hence, it can be assumed that exercise training should act as an important cornerstone of rehabilitation in Long- and Post-COVID to counteract both symptom burden and the loss of independence through decreased physical capacity. Initial studies, which investigated the influence of exercise training on the symptoms of those affected by long-term sequelae following a COVID-19 infection also showed positive effects; regular training supervised by appropriately trained exercise professionals and offered with an individualized and collaboratively coordinated increase in intensity reduced the severity of symptoms and increased physical and exercise capacities [17]. However, questions regarding the necessary intensity and frequency of the training as well as the necessary duration of the intervention remain unanswered. In addition, direct differences in the course of the intervention with regard to Long-COVID severity and sex-related differences have not yet been analyzed in detail.

Long-COVID is a new disease which affects a large number of people and can challenge the healthcare system. Affected patients and also health professionals need an empirically tested framework for the time-efficient conceptualization of individualized exercise interventions, which reflects the importance of collaboratively designed training to avoid the deterioration of symptoms by overtraining. Furthermore, professionals working in this field need practical tools to monitor progression during such interventions in Long-COVID patients. This study aims to test such a framework on Long-COVID patients with several objective (one-minute sit-to-stand test (1MSTST), workload) and patient-reported (FACIT–Fatigue score, Post COVID Functional Status scale) outcomes, taking into account the aforementioned aspects.

## 2. Materials and Methods

### 2.1. Ethical Approval, Recruitment, and Study Cohort

This study was approved by the ethical review board of the German Sport University Cologne (Reference Number 171/2022). Written informed consent was obtained from all participants. The recruitment was conducted using flyers and advertisements on the homepage of the S.P.O.R.T Institute and the German Sport University Cologne. A U09.9 diagnosis (“Post COVID-19 condition, unspecified”) from a medical doctor was required. There was no age limitation. The exclusion criteria included a rating of 4 on the Post COVID Functional Status Scale (PCFS) [18] and a planned inpatient stay within the first six weeks of the intervention.

Sixty-seven participants met the inclusion criteria, with twenty-two withdrawing and data from four missing. A detailed overview of the participants’ demographics as well as their medical history and SARS-CoV-2-specific information is illustrated in Table 1. A significant portion (73.2%, n = 30) reported at least one comorbidity, with the most common being endocrine, nutritional, or metabolic diseases (36.6%, n = 15). Analgesics were the most frequently reported medication (51.2%, n = 21). Fatigue-related symptoms (100%, n = 41) were the most common, followed by cognitive/neurological symptoms (87.8%, n = 36). According to the Post COVID Syndrome (PCS) Score [6], 61% (n = 25) were moderately affected and 39% (n = 16) were severely affected.

### 2.2. Study Design and Intervention

This study followed a single-arm pre-test-post-test design over an initial 12 weeks of the ongoing TRIBAL intervention. The evaluation focuses on time-related outcomes before the commencement of the initial training (T0), after six weeks (T1), and after 12 weeks (T2). Changes in the time-dependent results were also analyzed according to the following: (1) difference in moderate vs. severe LC, (2) difference in the frequency of training sessions, (3) difference in sex.

### 2.3. Testing Protocol

The testing included patient-reported outcomes (PROs) and objective measurements. The PROs comprised the post-COVID Functional Status (PCFS) scale [18] and the FACIT–Fatigue scale [19]. Objective measurements to assess exercise capacity included the one-minute sit-to-stand test (1MSTST) and workload (average wattage during a 15-minute session with a self-determined pace, derived via the KEISER M Series application) assessment. Vital parameters (blood pressure, heart rate, and oxygen saturation) were controlled during the testing sessions. The rating of perceived exertion (RPE) was gauged using a 10-point visual scale.

### 2.4. Exercise Intervention

The exercise sessions were supervised by exercise professionals or physiotherapists, specially trained and certified by Long-COVID Network Rhein-Neckar.

The reporting of the intervention was conducted according to the Consensus on Exercise Reporting Template (CERT) checklist [20].

The participants were asked to train at least twice weekly. Each exercise session consisted of 30 min. The *first part* always consisted of 15 min, and the *second part* followed a short break and covered the remaining 10–15 min. Each session was performed in groups of up to three people, with every participant receiving an individualized training regimen.

In the *first* part, the participants were asked to train on the M3i Total Body Trainer (Keiser GmbH, Coburg, Germany). This device was selected due to its medical approval in Germany, which ensures compliance with established health and safety standards. Additionally, it can be calibrated and verified for accuracy, further enhancing its reliability for use in clinical and research settings. The Total Body Trainer (TBT) combines an indoor bike with a cross trainer, which allows both the upper and lower extremities to be simultaneously trained. The primary goal was to train on the TBT for 15 min within a range of 50–60 rounds per minute (RPM). There was no minimum requirement in watts, to avoid overexertion. The initial intensity was estimated based on the individual performance and feedback in the baseline tests and expressed as the gear (resistance) on the TBT. Moderately affected participants started with a medium resistance (e.g., gear 11, where 60 RPM corresponds to about 60 watts), while the more severely affected participants started with a low resistance (e.g., gear 5, where 60 RPM corresponds to about 30 watts).

The progression of the intensity during the study period was based on three criteria: (1) if the participants easily met the time target (15 min) and increased the speed to 60 RPM and beyond within the respective resistance/gear, (2) if the reports of previous sessions did not include the worsening of symptoms, and (3) when the participants reported that they could tolerate a higher intensity. The intensity was then increased by switching up the gear of the TBT, starting with the last two minutes of the exercise session (13–15) and progressing by time during the following exercise sessions (11–15 min, then 7–15 min, and eventually 15 min). The average wattage (workload) of the first part was noted after every single exercise session.

After a short break, in the *second part* (the remaining 10–15 min), the participants were instructed to perform breathing exercises or low-intensity strengthening, stretching, or mobility exercises mainly using their body weight or low-resistance devices (e.g., TotalGym RS Encompass PowerTower (TotalGym Europe B.V, Hoofddorp, The Netherlands), rubber bands, foam rolls, kettlebells, or elastic balls) depending on their capacity and (e.g., orthopedic) demands. In the first weeks of the intervention, breathing and low-intensity stretching or mobility exercises were instructed to increase the participants’ body awareness. In the later course of the study, strengthening exercises were applied. To control the intensity and avoid overexertion throughout the *second part*, participants were asked to rate their perceived exertion and not exceed 6 on a 10-point scale. When feeling too exhausted, the participants also had the possibility to receive heat applications (heat therapy) or simply rest for the remaining time.

### 2.5. Data Analysis

Data analysis was conducted using IBM SPSS Statistics (Version 29). The normality was assessed using the Shapiro–Wilk test, with *p* > 0.05 indicating normally distributed data. The boxplots were visually inspected, and outliers were retained unless attributed to data entry errors. Before running each analysis, the assumptions were tested. Violations prompted corrections (e.g., Greenhouse–Geisser correction) for one-way repeated measures ANOVA [21] or the use of non-parametric alternatives. Significance level for all tests was set at *p* < 0.05.

Given the substantial number of analyses, only statistically significant results will be presented in detail in the results section. Same applies for effect sizes, which are reported only for statistically significant results. Effect sizes are reported as recommended, namely as Cohen’s *f* [22] for (one-way and one-way repeated measures) ANOVA, Kendall’s w for the Friedman test, $\eta^{2}$ for the Kruskal Wallis H-test [23], and Cohen’s d for independent samples *t*-test [24]. Non-significant findings are briefly summarized with the test results provided in Appendix A.

Statistical analyses encompassed patient-reported and objective outcomes, focusing on time, sex, training sessions, and symptom severity.

(a) Effects of time and sex: Outcome values are reported as mean (SD). A one-way repeated measures ANOVA assessed differences in the FACIT–Fatigue, 1MSTST, and workload scores across three time points (T0, T1, and T2) during the 12-week intervention. Post hoc tests reported the mean differences (M.) with 95% confidence intervals (CI). If the normality was violated or for ordinal outcomes (PCFS), a Friedman test with median (Mdn.) scores was used. To investigate the interaction of time and sex, a two-way mixed ANOVA was employed. (b) PCS score and change scores: Differences in change scores (Δ₂) between moderately and severely affected groups (based on the PCS scores) were analyzed using independent *t*-tests or Mann–Whitney U tests, with mean differences (M.) and 95% CI reported for *t*-tests. (c) Completed Training Sessions (CTS) and change scores: Correlation analyses examined the relationship between CTS and change scores (Δ₂) in the FACIT–Fatigue, 1MSTST, and workload scores. Pearson’s or Spearman’s correlations were applied based on the distribution. Participants were divided into three groups by CTS (<21, 21–24, >24). A one-way ANOVA or Kruskal–Wallis H test assessed differences in outcome changes between the groups. (d) Relationships between the outcomes: Correlations between PCS, PCFS, FACIT–Fatigue, 1MSTST, and workload scores at T0 were analyzed using Pearson’s or Spearman’s correlations.

## 3. Results

The summarized results related to the graphs are presented below, and the outcomes that did not reach statistical significance are described in Appendix A and Appendix B.

### 3.1. Effects of Time

**Figure 1:**
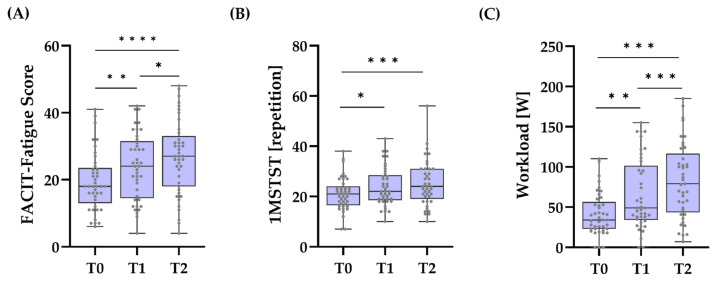
Changes in the Fatigue score and exercise capacity over time. The figure shows boxplots illustrating the distribution of the (**A**) FACIT–Fatigue scores, (**B**) 1MSTST repetitions, and (**C**) workload scores across the three testing sessions (T0, T1, and T2). The results highlight improvements in all three parameters over time. The box represents the interquartile range (IQR), with the median denoted by the horizontal line inside the box. Each dot represents one individual. Whiskers extend from the box to the minimum and maximum values within 1.5 times the IQR. Statistically significant differences between the different time points, as calculated with post hoc pairwise comparisons following the Friedman test, are highlighted with asterisks. * *p*< 0.05; ** *p* < 0.01; *** *p* < 0.001. **** *p* < 0.0001. n = 41.

The FACIT–Fatigue scores significantly increased from T0 to T1 (M. = −4.56, 95% CI [−7.57, −1.55], *p* = 0.002), from T0 to T2 (M. = −7.41, 95% CI [−11.02, −3.81], *p* < 0.001), and from T1 to T2 (M. = −2.85, 95% CI [−5.49, −0.22], *p* = 0.03). The effect size was large: Cohen’s *f* = 0.67 (Figure 1A).

The 1MSTST scores differed significantly over time: $\chi^{2}$(2) = 19.35, *p* < 0.001. Effect size was small: Kendall’s w = 0.24. Median values increased from T0 (Mdn. = 21) to T1 (Mdn. = 22) to T2 (Mdn. = 24). Post hoc tests showed that T0 and T1 (z = −0.65, *p adjusted* = 0.01) and T0 and T2 (z = −0.93, *p adjusted* < 0.001) differed significantly, but T1 and T2 did not (Figure 1B).

Statistical significance was also established in the differences between workload scores: $\chi^{2}$(2) = 62.27, *p* < 0.001. Effect size of the differences between time points was large: Kendall’s w = 0.76. Median values increased from T0 (Mdn. = 34) to T1 (Mdn. = 49) to T2 (Mdn. = 79). There were statistically significant differences between T0 and T1 (z = −0.829, *p adjusted* = 0.001) and T0 and T2 (z = −1.73, *p adjusted* < 0.001), as well as between T1 and T2 (z = −0.90, *p adjusted* < 0.001) (Figure 1C).

### 3.2. Effects of Sex

A two-way mixed ANOVA yielded no significant interactions between time and sex, neither for the FACIT–Fatigue nor for 1MSTST or workload scores. Additionally, the main effect of group did only reveal statistically significant differences between males and females for orkload scores (F (1,39) = 4.39, *p* = 0.043, partial $\eta^{2}$ = 0.10), with males scoring significantly higher than females. The effect size for the main effect of group was medium: Cohen’s *f* = 0.33 (Appendix A.1).

### 3.3. Effects of Training Sessions

**Figure 2:**
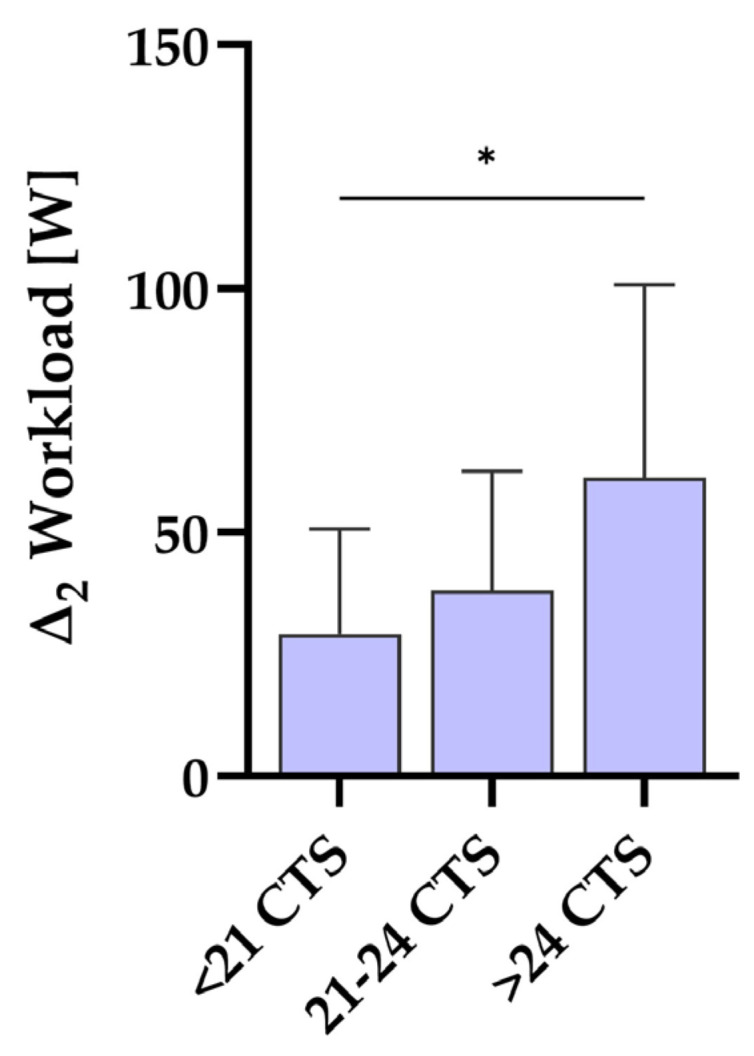
Difference in workload score between 12 weeks and 0 weeks related to number of complete training sessions (CTS). Participants that completed more than 24 CTS showed higher increment in workload score after 12 weeks of training compared to participants that completed less than 21 CTS. * *p* < 0.05. Participant count for groups is as follows: <21 CTS with n = 13, 21–24 CTS with n = 17, and >24 CTS with n = 11.

Results indicated a significant difference in workload scores across the complete training sessions (CTS) groups, F(2, 40) = 3.96, *p* = 0.027, with significant differences between group 1 (<21 sessions) and group 3 (>24 sessions) (M = −32.03, 95% CI [−60.52, −3.54], *p* = 0.025), emphasizing a significantly greater increase in the workload scores for participants who completed more than 24 compared to those who completed less than 21 training sessions (Figure 2; Appendix A.2 and Appendix Table A2). Analysis further revealed no significant effect between number of CTS and difference in 1MSTST or difference in FACIT–Fatigue score, respectively (Appendix A.2 and Appendix Table A2).

### 3.4. Effects of Symptom Severity

#### 3.4.1. Change in PCFS Score After 12 Weeks of Intervention

The median PCFS score (3) did not change significantly during the intervention. Additionally, the distribution of the scores (65.85% scoring 3 pre-intervention and 60.98% scoring 3 post-intervention) indicated moderate functional limitations for the majority of the participants before and after the twelve weeks of the intervention.

#### 3.4.2. Change in Workload, Fatigue Score, and 1MSTST After 12 Weeks Related to Initial PCS Score

**Figure 3:**
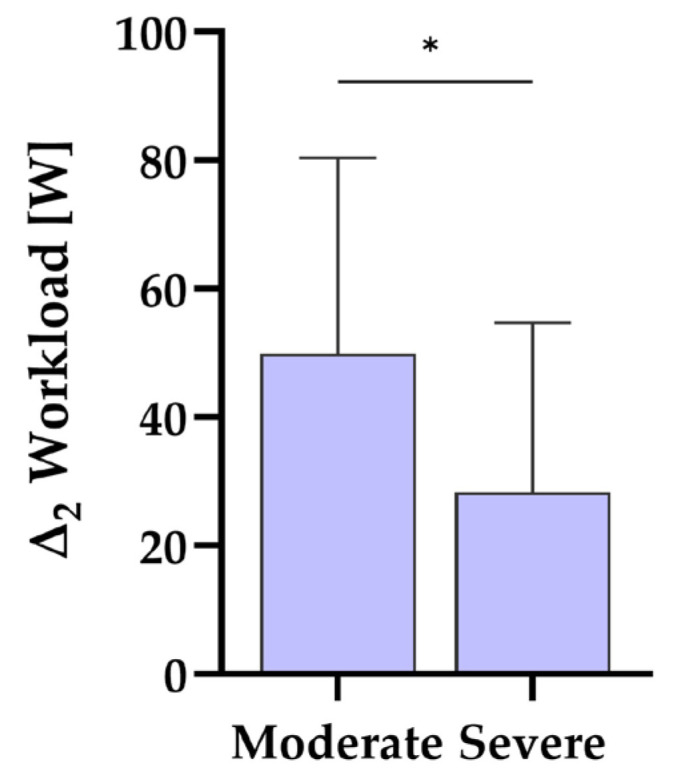
Difference in workload score between 12 weeks and 0 weeks related to PCS score. Statistically significant differences between groups, as calculated by post hoc tests following a one-way ANOVA, are highlighted with an asterisk. * *p* < 0.05. Participant count for groups is as follows: severe (>26.25) with n = 16, and moderate (10.75–26.25) with n = 25.

The $\Delta_{2}$ values were examined for moderately and severely affected groups, classified by PCS scores. Analysis revealed significant differences in $\Delta_{2}$ of workload scores between moderately and severely affected participants (M = −23.60, 95% CI [−5.30, −41.91], t(39) = −2.61, *p* = 0.013), indicating a larger increase in workload scores for moderately affected participants (Figure 3; Appendix A.3 and Appendix Table A2). The Fatigue score and 1MSTST increased comparably in both groups. The groups did not significantly differ (see Appendix A.3 and Appendix Table A2).

### 3.5. Correlation Analyses

Correlation analyses showed no significant relationships between CTS and $\Delta_{2}$ for any outcome (Appendix A.3. The results indicated a correlation between lower PCFS scores and higher FACIT–Fatigue scores, as indicated by a moderate negative correlation ($r_{s}$ = −0.47, *p* = 0.002). Higher FACIT–Fatigue scores correlated with higher workload scores, as indicated by a moderate positive correlation ($r_{s}$ = 0.42, *p* = 0.007), and higher workload scores correlated strongly with higher 1MSTST scores, as indicated by a strong positive correlation ($r_{s}$ = 0.50, *p* < 0.001) (Table 2).

## 4. Discussion

Twelve weeks of individualized and controlled exercise training, combining one part of endurance training on a stationary Total Body Trainer with another part of mixed breathing, stretching, and/or strengthening exercises, led to significant improvements in exercise capacity (as measured by 1MSTST and workload test) and fatigue (as measured by FACIT–Fatigue scale) in male and female ambulatory Long-COVID patients. The controlled approach of the supervision by exercise professionals and a criteria-based collaborative progression of the intensity led to improvement rates of 95.1% in the workload and 82.9% in both the FACIT–Fatigue score and 1MSTST over twelve weeks, showing the success of the program for most of the participants.

### 4.1. Main Effect of Time

These results are in line with prior investigations examining the influence of exercise training on fatigue and exercise capacity in several other chronic conditions including cancer and multiple sclerosis [14,16]. The current study, furthermore, adds evidence to early empirical works finding reduced fatigue and enhanced exercise capacity after different exercise training regimens in Long-COVID patients [25].

The sample analyzed in the present study presented with a high symptom and fatigue burden as well as restrictions in exercise capacity and daily functioning. More precisely, almost half of the participants (41.5%) were classified as severely affected according to the PCS score, and mean FACIT–Fatigue scores were below a value of 30 and thus clinically relevant across all time points [6,26,27]. Moreover, the mean 1MSTST score was lower and the median PCFS scores were higher compared to a previous study in Long-COVID patients [17].

Regarding fatigue symptomatology, every single participant investigated in this study reported fatigue-related symptoms prior to the intervention. Furthermore, despite a clinically important mean change by 7.42 points in the FACIT–Fatigue scores over the course of the intervention, the mean score remained clinically elevated after the twelve weeks (26.80 points), considering an increase by three to five points and a cut-off score of ≤30 as clinically relevant [27,28]. For comparison, another study which also used the FACIT–Fatigue score in a cross-sectional investigation of symptomatic individuals nine months after the acute infection reported a mean score of 39.2 points [26]. The finding that fatigue symptoms were still in a clinically relevant range after the twelve-week program despite the participants improving significantly suggests that the training needs to be continued to bring the values into line with a healthy reference group.

The impact of the symptom burden was also expressed in the second patient-reported outcome, that was assessed in this study, the PCFS score. The median PCFS score (3) did not change significantly during the intervention. Additionally, the distribution of the scores as reported in Section 3.4.1 indicated moderate functional limitations for the majority of the participants before and after the twelve weeks of the intervention. While the improvement in fatigue is in line with previous studies investigating the effect of exercise training in Long-COVID patients, the stagnation in the PCFS scores might contrast to previous findings [17]. However, in one study cited by Sick & König [17], reported improvements were expressed as means [29], while the present study statistically analyzed median values as recommended for ordinal scales such as the PCFS scale [18]. Another study by the same author derived improvements in PCFS scores by comparing the number of participants presenting with a score of <2 before and after the intervention [30]. Another study reported an improvement in the median PCFS score over six weeks [31]. The different findings in this study could be explained by two reasons: First, the concerned study investigated a multidisciplinary outpatient rehabilitation, including nutritional education and psychosocial counseling besides structured exercise training. Second, the baseline median score was 2 in the examination of Nopp et al. [31], indicating fewer functional limitations compared to the sample analyzed in this study (median of 3) and thus possibly indicating a better prognosis with regard to the potential improvements during the intervention related to the initial impairments.

The differences between the studies regarding the severity of the functional limitation are also reflected in the lower exercise capacity of the patients examined in the present study. While Nopp et al. [31] reported a mean of 33.3 repetitions at baseline testing in the 1MSTST, the sample of this study performed a mean of 20.98 repetitions before and 25.07 repetitions after twelve weeks. Although the score increased by a mean of 4.10 repetitions, which exceeds the minimal important difference (≥3 repetitions) according to [32], it remained comparably low. This finding adds to the results previously reported for the FACIT–Fatigue scores and implicates restrictions in exercise capacity.

As a second outcome depicting exercise capacity, workload scores improved significantly over time, with a mean increase of 24.50 watts in the first six weeks and of 43.03 watts over the twelve weeks. The workload scores derived in this study are hardly comparable to other investigations of exercise interventions since the assessment is not standardized as in questionnaires (like the FACIT–Fatigue) or standardized tests like the 1MSTST. The present study defined workload as the average wattage of 15 min on the Total Body Trainer, which allowed the simultaneous training of the lower and upper extremities and therefore differs from exercise training on a stationary bicycle ergometer by involved muscles and metabolic demands. Comparable studies did either not specify which device was used for endurance training [29,30,31] or reported the use of a bicycle ergometer [25]. Additionally, studies mentioning workload as an outcome either did not specify training time at all [31] or differed in training time (e.g., in [25], participants were asked to train for 18 min). The use of different devices and different definitions to assess the workload hinders their comparability with the present study.

However, the improvements in workload are in line with the improvements in FACIT–Fatigue score and 1MSTST and therefore reflect the general trainability of Long-COVID patients and the positive influence of training on one of the main symptoms in Long-COVID patients, fatigue, as well as exercise capacity. These findings complement previous studies reporting an amelioration of symptoms as well as physical performance parameters [17].

Based on these results, it could be hypothesized that ongoing training exceeding the twelve weeks, which have been investigated in this study, would lead to further improvements in fatigue symptomatology as well as in exercise capacity, which eventually would also reduce functional restrictions in daily living (PCFS score). The development of improvements, which does not only present in the mean scores but also in the proportion of the participants experiencing ameliorations (of at least one point) after six weeks (78% in FACIT–Fatigue, 70.7% in 1MSTST, 87.8% in workload score, and 4.9% in PCFS score) and after twelve weeks (82.9% in FACIT–Fatigue, 82.9% in 1MSTST, 95.1% in workload score, and 14.6% in PCFS score) supports this hypothesis. This hypothesis is further supported by previous investigations which showed improvements in several outcomes but were limited to a total duration of four to twelve weeks [17].

Further, it should be noted that 14.6% (n = 6) of the participants in the analyzed sample showed decreased FACIT–Fatigue scores, 17.07% (n = 7) showed reductions in 1MSTST, and 4.9% (n = 2) showed reductions in workload scores after twelve weeks. Following the line of basic training principles [33], one might suggest that the participants that showed a worsening in the previously stated scores did not set sufficient training stimuli for (physiological or psychological) adaptations or that the total duration of the intervention was not long enough. To support this assumption, individual analyses revealed that the two participants showing reductions in their workload scores after 12 weeks only completed 15 and 24 training sessions, respectively. The individuals showing decreased FACIT–Fatigue scores completed less than 21 (n = 3) or between 21 and 24 (n = 3) training sessions. And, finally, n = 4 participants with CTS between 21-24 and n = 3 participants with CTS > 24 showed reduced 1MSTST scores. Yet, because the highest increment in the workload score was achieved by a number of participants with CTS > 24, it is concluded that Long-COVID patients benefit from regular training with two to three training sessions per week.

However, the low number of CTS cannot completely explain the described findings, as further explained below. Previous studies reported that a substantial number of Long-COVID patients share symptoms with ME/CFS, in particular post-exertional malaise (PEM), which describes the deterioration of symptoms after (physical or mental) activity [34,35]. It could be hypothesized that some of the participants might also fit the clinical definition of ME/CFS, but the presence of ME/CFS was not systematically assessed. However, since the same participants did not consistently show the reductions mentioned, a more detailed analysis is required here, which also includes other factors.

### 4.2. The Effect of Sex

Although a “sex bias” is discussed in the literature, higher prevalence rates and higher symptom severity have been reported in women [3,36]. Based on the findings of this study, this bias does not translate into any differences in trainability between both sexes, as indicated by the absence of interaction effects in the two-way mixed ANOVA. This is in line with Mooren et al. [25] who did not find a different response to training with respect to sex. It can be stated that both sexes did benefit similarly from the individualized training approach conducted in this study. Therefore, sex is not a characteristic that requires differentiation in training management. However, it should be noted that the present study included a small sample size. Future investigations including more participants should further validate these findings.

The only statistical difference that was found between the sexes was in the mean workload score, which was higher in males than in females when averaged across all testing sessions. This result is in line with a study in cardiorespiratory outpatient rehabilitation that showed a reduced VO_2_ peak in females compared to males, both before and after exercise training [37], but this was not related to Long-COVID or the training applied herein. The (biological) mechanisms which are discussed to be responsible for the differences in mean workload production between males and females are beyond the scope of this investigation and are therefore not further discussed.

### 4.3. The Influence of Symptom Severity

To date and knowledge of the authors, this is the first study implementing the PCS score in an investigation of exercise training. At baseline, 41.5% of the participants were classified as severely affected, which adds to the previously discussed evidence of a comparably high disease burden in the present sample. For comparison, Bahmer et al. [6] found 13–20% of Long-COVID patients to be severely affected in the initial study, in which the PCS score was introduced. Furthermore, none of the participants investigated in this study were classified as only mildly influenced.

The investigation of differences between moderately and severely affected participants showed significant differences for the development of the workload, yet the scores did improve in both groups, namely by a relative mean of 77.8% in severely affected and of 113.6% in moderately affected participants. Furthermore, FACIT–Fatigue and 1MSTST scores developed comparably in both groups, showing increments in the mean scores over the course of the intervention. These findings indicate that, despite the difference in workload scores, both groups were trainable and experienced ameliorations in fatigue symptomatology and exercise capacity. As discussed previously in comparing male and female participants, it appears that both moderately and severely affected PCS patients improve through exercise training.

It was beyond the scope of this analysis to systematically assess which characteristics could be responsible for the differing workload production in severely affected Long-COVID patients. Since this is the first study comparing the results for Long-COVID patients based on a symptom severity score, the results remain preliminary. It would be of high clinical relevance to clarify the findings of this study with a reasonable sample size to further evaluate if there are differences in the adaption between patient groups differing in symptom severity.

However, deducing practical considerations for training management in Long-COVID patients from the findings of this study, it appears most important to adhere to the basic training principle of individualization in order to reflect the conditions of patients with varying degrees of severity and to be able to address these in training planning [33]. The principle of individualization is in line with the recommendations as well as the prior investigations of exercise training in this cohort [10,17].

### 4.4. The Influence of Completed Training Sessions

The number of completed training sessions (CTS) did influence the change scores only in one comparison in the workload, showing no consistent dose–response relationship between the training frequency and adaptions in fatigue symptomatology or exercise capacity in the studied Long-COVID sample. Consistent with this finding, Mooren et al. [25] did not find an effect of overall training sessions on outcomes.

The finding of a differing change in workload scores between the group with the most training sessions compared to the group with the least training sessions could be explained by a more pronounced cardiovascular response in relation to a higher number of training sessions. However, the positive mean in change scores across the different ranges of the training sessions in fatigue and exercise capacity (refer to Appendix B, Table A2) points to the assumption that the health benefits experienced by the participants of this study were mostly independent of the training frequency. Nevertheless, according to the principles of training science, it should be taken into account that a certain number of training stimuli are necessary to achieve optimal adaptations [33].

While previous studies have recommended that participants train three to five times per week, with investigation periods of four to twelve weeks [17], the present study recommends at least two training sessions per week, which was met only by 26.8% of the participants (n = 11). Furthermore, the range of the completed training sessions (15–36 CTS) over the twelve weeks indicated a high variability in the adherence to the recommendations. The participant with the most training sessions trained three times per week, while a mean of 1.25 sessions per week accounted for the participant with the least training sessions. The main self-reported reasons for the lower numbers of attended training sessions included scheduling problems, personal problems (job/family), illness, or other.

To summarize, finding no consistent dose–response relationship, training frequency does not appear to be the sole limiting factor for the positive effects observed across the different ranges of CTS across the twelve weeks investigated in this study.

### 4.5. Relationships of Outcomes

The findings of correlation analyses offer valuable insights into the baseline dynamics of Long-COVID patients undergoing exercise training. The negative correlation between the PCFS and FACIT–Fatigue scores suggests that patients with a lower functional status may experience lower levels of fatigue, underscoring the intricate relationship between functionality and subjective well-being which has already been stated in previous investigations [38,39].

The positive correlation between the FACIT–Fatigue and workload scores implies that patients being less fatigued may be capable of more demanding exercise training. This finding appears to be in line with the finding of the present study that the FACIT–Fatigue and workload scores both increased significantly across the different testing sessions. Contrary to this assumption, a relationship between the workload and 1MSTST scores was found which was not expressed as an equal improvement in both values: while the workload differed significantly between all three time points in the time-related analyses, 1MSTST scores did not increase significantly between six and twelve weeks.

The 1MSTST primarily measures lower limb strength endurance (quadriceps, hamstrings, and gluteal muscles) during a functional task (sit-to-stand) and progressively increases cardiovascular demands through elevated heart rate and oxygen consumption (VO_2_max), engaging both aerobic and anaerobic systems. In contrast, the workload measurement used in this study emphasizes aerobic capacity and involves whole-body coordination, requiring simultaneous alternating movements of the arms and legs. It could be assumed that the workload measure may be more sensitive to exercise-induced changes in aerobic capacity, as the 1MSTST improvements between 6 and 12 weeks were not statistically significant. However, as summarized by Sick & König [17], most of the studies investigating exercise training in Long-COVID patients report improvements in physical performance (including several versions of the sit-to-stand test) as well as fatigue measures.

## 5. Conclusions

This study demonstrates that the twelve-week individualized and controlled exercise program, combining endurance training with breathing, stretching, and strengthening exercises, demonstrated significant improvements in fatigue and exercise capacity among ambulatory Long-COVID patients. The intervention’s controlled approach, supervised by exercise professionals, yielded high improvement rates in the workload (95.1%) and both the FACIT–Fatigue and 1MSTST (82.9%) scores. Despite improvements, fatigue symptoms remained clinically relevant, emphasizing the need for continued training. This study explored sex differences, indicating a similar trainability for both sexes, and considered the influence of symptom severity, showing positive outcomes for both moderately and severely affected Long-COVID patients. Overall, this study contributes valuable insights into the effectiveness of individualized exercise interventions for Long-COVID patients and highlights areas for future exploration and refinement in the management of Long-COVID symptoms including the need for longer-term interventions to fully assess the potential benefits of exercise on recovery outcomes. Further research should give attention to factors influencing the adherence and individual responses to training in terms of symptom severity and include monitoring of other common symptoms.

## Figures and Tables

**Table 1:** Baseline characteristics.

Variable	Value
Age [years]	48.05 ± 15.15
BMI [kg/m^2^]	26.44 ± 5.36
Sex [women]	24 (58.5%)
**Comorbidities**	
Reported comorbidities [yes]	30 (73.2%)
Endocrine, nutritional, or metabolic diseases	15 (36.6%)
Diseases of the musculoskeletal system and connective tissue	12 (29.3%)
Diseases of the cardiovascular system	9 (21.9%)
Diseases of the respiratory system	8 (19.5%)
Pain disorders/chronic pain syndrome	5 (12.2%)
Neurological diseases	3 (7.3%)
Psychiatric disorders	3 (7.3%)
Others ^1^	4 (9.7%)
**Medication**	
Analgesics	21 (51.2%)
Thyroid medication	9 (21.9%)
Antidepressants	9 (21.9%)
Antihypertensives	8 (19.5%)
Corticosteroids	7 (17.1%)
Anticoagulants	5 (12.2%)
Bronchodilators	4 (9.7%)
Others ^2^	9 (21.9%)
**SARS-CoV-2 specific information**	
Duration between infection and training start [weeks]	51.54 ± 43.10
Vaccination status [yes]	41 (100%)
Received two SARS-CoV-2 vaccinations	7 (17.1%)
Received three SARS-CoV-2 vaccinations	29 (70.7%)
Received four SARS-CoV-2 vaccinations	5 (12.2%)
Inpatient treatment since SARS-CoV-2 infection [yes]	14 (34.1%)
**Post COVID-19 specific information**	
Number of symptom clusters ^3^ per patient, median	4
Fatigue	41 (100%)
Cognitive/neurological system	36 (87.8%)
Chest symptoms	26 (63.4%)
Musculoskeletal system	24 (58.5%)
Anxiety, depression, or sleep disorders	16 (39%)
Tachycardia	8 (19.5%)
Gastrointestinal symptoms	6 (14.6%)
Flu-like symptoms, chills, or fever	6 (14.6%)
Others ^4^	3 (7.3%)
PCS score ^5^	23.59 ± 7.33
None/mild	0
Moderate	24 (58.5%)
Severe	17 (41.5%)

^1^ Other comorbidities included removal of gallstone (n = 1), infection with the Epstein–Barr virus (n = 1), borreliosis (n = 1), and hyperuricemia (n = 1). ^2^ Other medications included antipsychotics (n = 1), proton pump inhibitors (n = 1), sedatives (n = 2), alpha blockers (n = 1), anticonvulsants (n = 1), uricostatics (n = 1), TNF (tumor necrosis factor) blockers (n = 1), and non-steroidal anti-inflammatory drugs (n = 1). ^3^ Symptoms were clustered referring to [3] as follows: fatigue, including fatigue-related symptoms such as a state of exhaustion, reduced exercise tolerance, and prolonged recovery time. Cognitive/neurological symptoms included brain fog, concentration and memory problems, smell or taste distortion, vertigo, dizziness, balance problems, tremor, sound and light sensitivity, headaches/migraines, and vision impairments. Chest symptoms included shortness of breath, chest pain, and wheezing. Musculoskeletal symptoms included muscle and joint pain, paresthesia, muscle cramps, and muscle weakness. Gastrointestinal symptoms included nausea, vomitus, and abdominal pain. Others included lymphoedema and hypotonia. ^4^ Other symptoms included urinary incontinence (n = 1), lymphoedema (n = 1), and hypotonia under exhaustion (n = 1). ^5^ The PCS score was calculated according to [6] with thresholds for the PCS score classification of 10.75 and 26.25 for none/mildly, moderately and severely affected participants, respectively.

**Table 2:** Correlation matrix of outcome variables at T0.

Variables	1.	2.	3.	4.
1. PCS	-	-	-	-
2. PCFS	0.08	-	-	-
3. Fatigue	−0.23	−0.47 **	-	-
4. 1MSTST	−0.29	−0.27	0.13	-
5. WL	−0.25	−0.30	0.42 **	0.50 ***

PCS = Post COVID Syndrome Score; PCFS = Post COVID Functional Status Scale; 1MSTST = one minute sit to stand test; WL = workload; ** *p* < 0.01, *** *p* < 0.001.

## Data Availability

The data that support the findings of this study are available upon reasonable request from the corresponding author. The data are not publicly available due to privacy or ethical restrictions.

## Appendix A

### Appendix A.1. Interaction Effects of Time and Sex

FACIT–Fatigue scores:

There was no statistically significant interaction between sex and time in the FACIT Fatigue scores, F(2, 78) = 0.83, *p* = 0.441, partial $\eta^{2}$ = 0.02. The main effect of the group showed that there was no statistically significant difference in the FACIT Fatigue scores between the sexes, F(1, 39) = 0.22, *p* = 0.643, partial $\eta^{2}$ = 0.01.

1MSTST scores:

There was no statistically significant interaction between sex and time in the STS scores, F(1.598, 62.334) = 0.43, *p* = 0.608, partial $\eta^{2}$ = 0.01, ε = 0.80. The main effect of the group showed that there was no statistically significant difference in the STS scores between the sexes, F(1,39) = 0.14, *p* = 0.714, partial $\eta^{2}$ = 0.003.

Workload (WL) scores:

There was no statistically significant interaction between sex and time in the WL scores, F(1.531, 59.707) = 1.99, *p* = 0.156, partial $\eta^{2}$ = 0.048, ε = 0.76. The main group effect showed that there was a statistically significant difference in mean WL scores between men and women, F(1, 39) = 4.392, *p* = 0.043, partial $\eta^{2}$ = 0.101. The effect size for the group differences was medium: Cohen’s *f* = 0.33.

### Appendix A.2. Completed Training Sessions (CTS) and Change Scores

#### Appendix A.2.1. Relationship Analyses

Spearman’s rank–order correlation indicated that the number of CTS did not correlate significantly with any of the outcomes, neither with the $\Delta_{2}$ of the FACIT–Fatigue scores, $r_{s}$ = −0.05, *p* = 0.597, n = 41, nor with the $\Delta_{2}$ of the 1MSTST scores, $r_{s}$ = −0.30, *p* = 0.052, n = 41, nor with the $\Delta_{2}$ of the WL scores, $r_{s}$ = 0.29, *p* = 0.069, n = 41.

#### Appendix A.2.2. Difference Analyses Related to Number of CTS

FACIT–Fatigue scores:

The mean rank of the FACIT–Fatigue change scores was not statistically significantly different between the groups < 21 CTS, 21–24 CTS, and >24CTS, $\chi_{2}$(2) = 1.21, *p* = 0.546 (two-sided).

1MSTST scores:

There were no statistically significant differences in the 1MSTST scores between the groups < 21 CTS, 21–24 CTS, and >24 CTS, F(2, 40) = 1.07, *p* = 0.51.

### Appendix A.3. Interaction Effects of PCS Score and Change Scores

FACIT–Fatigue scores:

There was no statistical significant difference in the Fatigue score changes between the groups, M. = −0.206, 95%CI [−6.198, 5.786], t(39) = −0.07, *p* = 0.945 (two-sided).

1MSTST scores:

The distributions of the change in the 1MSTST scores for the moderately and severely affected patients were similar. The median change in the 1MSTST scores was not statistically significantly different between the groups, U = 169.5, z = −0.92, *p* = 0.360.

### Appendix A.4. Relationships between Outcomes

Spearman’s rank–order correlation found a statistically significant moderate negative correlation between the PCFS scale scores and FACIT–Fatigue scores ($r_{s}$ = −0.47, *p* = 0.002), a moderate positive correlation between WL scores and FACIT–Fatigue scores ($r_{s}$ = 0.42, *p* = 0.007), and a strong positive correlation between the WL scores and 1MSTST scores ($r_{s}$ = 0.50, *p* < 0.001). Except for these, there were no statistically significant correlations.

The PCS scores did not correlate significantly with the PCFS scores ($r_{s}$ = 0.08, *p* = 0.638), FACIT–Fatigue scores ($r_{p}$ = −0.23, *p* = 0.154), 1MSTST scores ($r_{p}$ = −0.29, *p* = 0.061), or WL scores ($r_{s}$ = −0.25, *p* = 0.119). The PCFS scores did not correlate significantly with the 1MSTST scores ($r_{s}$ = −0.27, *p* = 0.087) or WL scores ($r_{s}$ = −0.30, *p* = 0.057). The FACIT–Fatigue scores did not correlate significantly with the 1MSTST scores ($r_{s}$ = −0.13, *p* = 0.399).

## Appendix B

**Table A1:** Overview of outcomes at time-related testing sessions.

	PCFS	Fatigue	1MSTST	WL
T0	3	19.39 (8.72) **^,^***	20.98 (6.59) *^,^***	41.29 (26.47) **^,^***
T1	3	23.95 (9.92) **^,^*	23.83 (7.59) *	64.98 (43.88) **^,^***
T2	3	26.80 (11.11) ***^,^*	25.07 (9.02) ***	82.76 (46.97) ***^,^***

This table shows the median for the PCFS and the mean (standard deviation) for the other outcomes pre-intervention (T0), at six weeks (T1), and at twelve weeks (T2). PCFS = Post COVID Functional Status Scale; 1MSTST = one-minute sit-to-stand test; WL = workload. Statistical significance is highlighted with asterisks, with * *p* < 0.05; ** *p* < 0.01; and *** *p* < 0.001. A comma (,) after the first asterisk indicates more than one statistically significant comparison; e.g., in the third column, the FACIT–Fatigue scores differed significantly in the following comparisons: T0 vs. T1 **, T0 vs. T2 ***, and T1 vs. T2 *.

**Table A2:** Overview of $\Delta_{2}$ of outcomes for the three groups of CTS and two groups of PCS.

	n	$\Delta_{2}$ Fatigue	$\Delta_{2}$ 1MSTST	$\Delta_{2}$ WL
CTS, Group 1	13	7.62 (12.57)	5.69 (6.57)	29.15 (21.57) *
CTS, Group 2	17	6.41 (7.48)	4.00 (5.61)	38.12 (24.46)
CTS, Group 3	11	8.73 (7.59)	2.36 (3.85)	61.18 (39.67) *
PCS, moderate	24	7.50 (8.68)	3.96 (7.06)	51.25 (30.38) *
PCS, severe	17	7.29 (10.22)	4.29 (2.39)	27.65 (25.71) *

Overview of participants and means (standard deviations) of $\Delta_{2}$ (T2-T0) values for the three groups of CTS and the two groups of PCS. CTS = completed training sessions; CTS group 1: <21 CTS; group 2: 21–24 CTS; 3: >24 CTS; PCS = Post COVID Syndrome Score; 1MSTST = one-minute sit-to-stand test; WL = workload. Statistical significance is highlighted with asterisks, with * *p* < 0.05.
